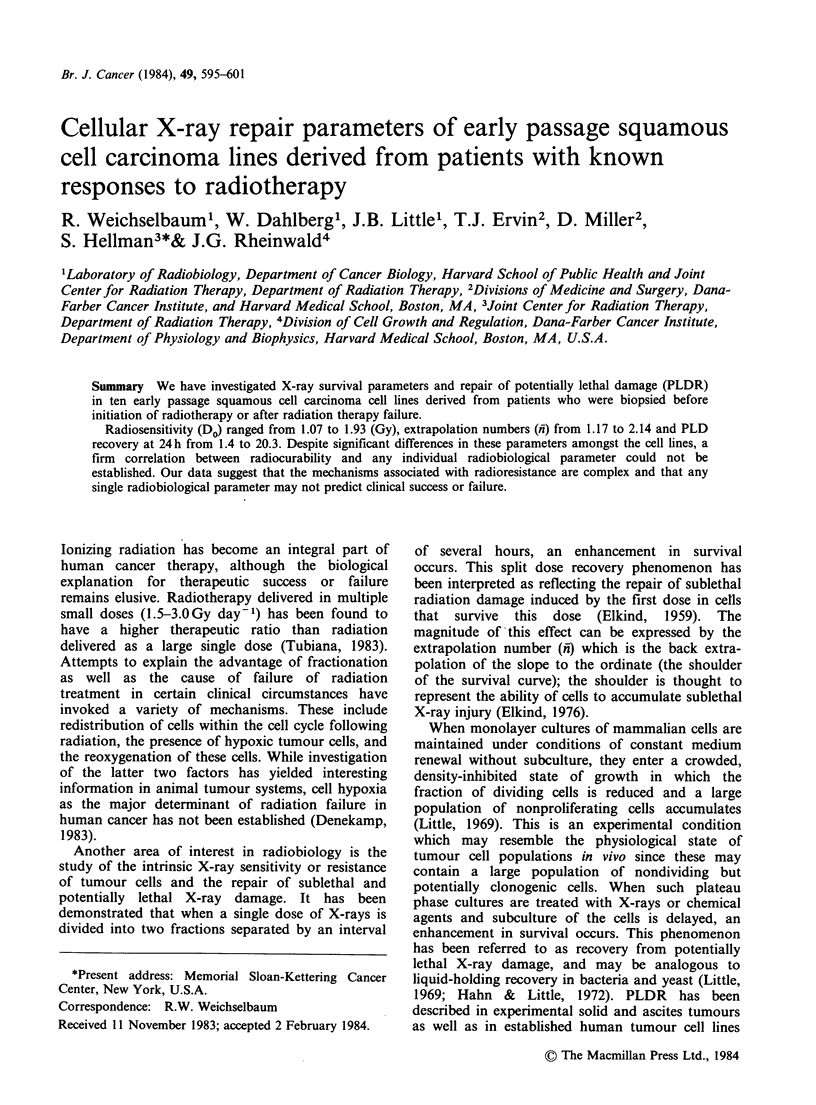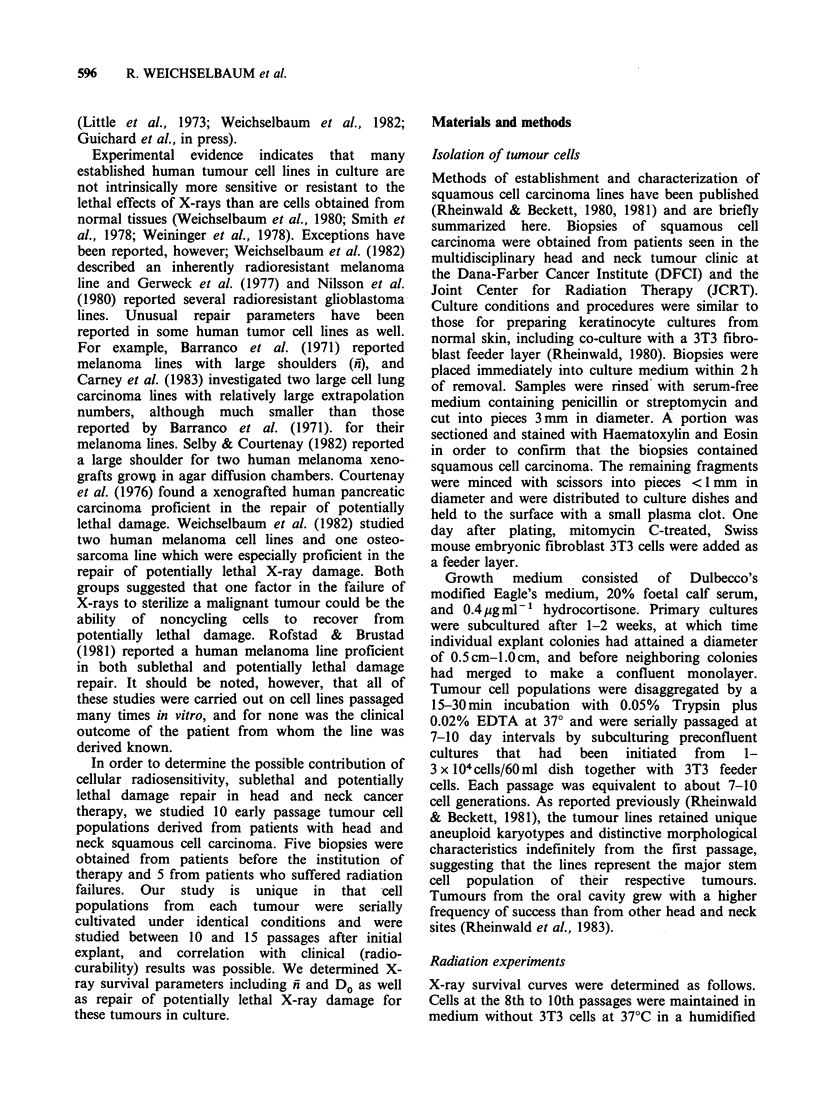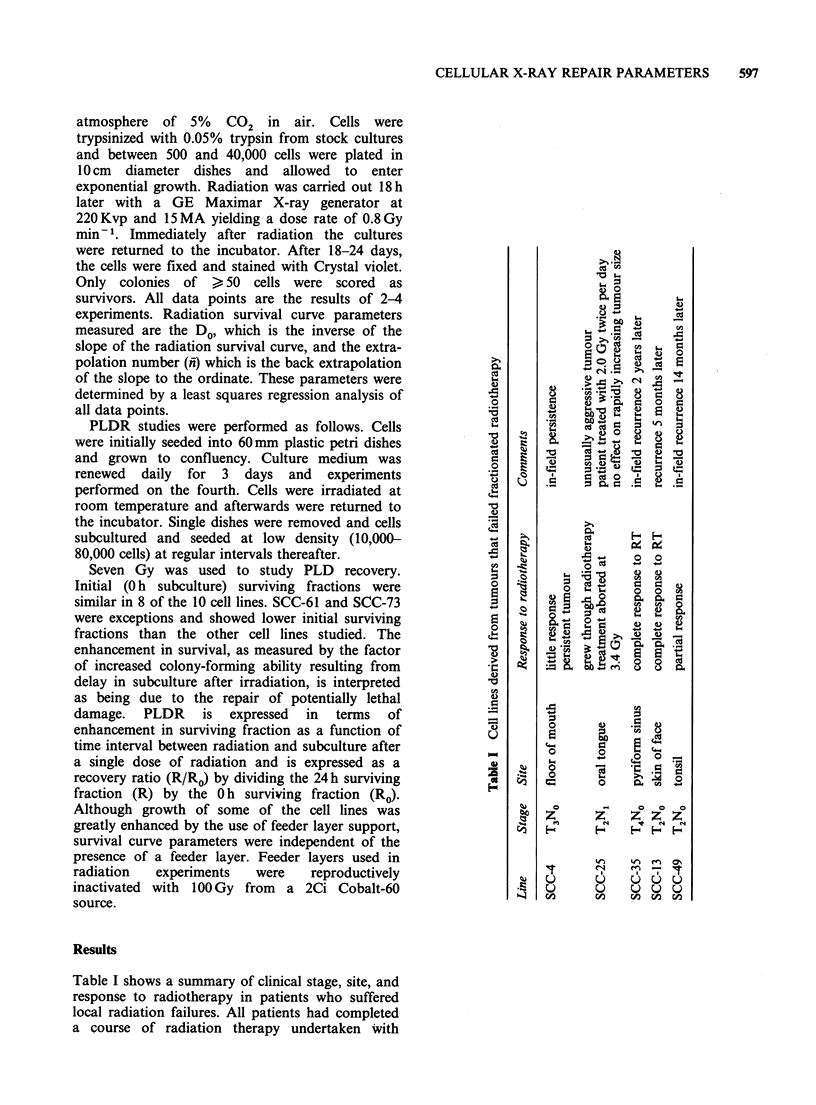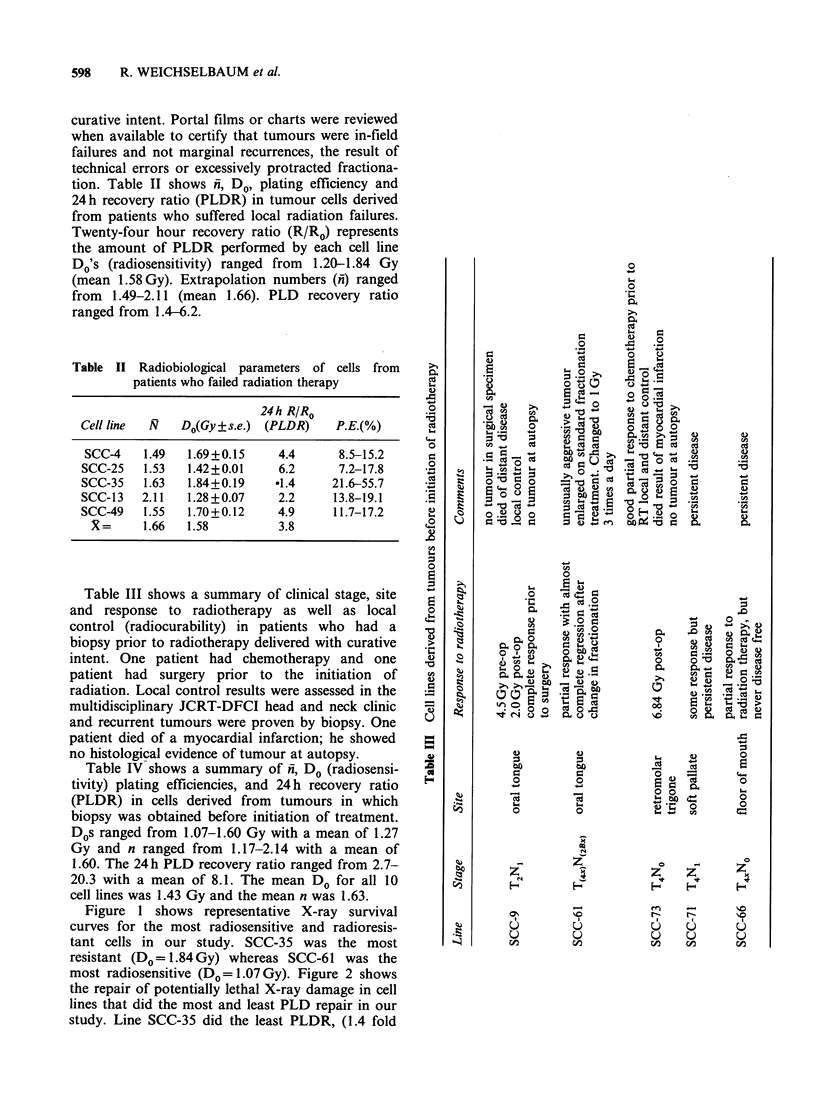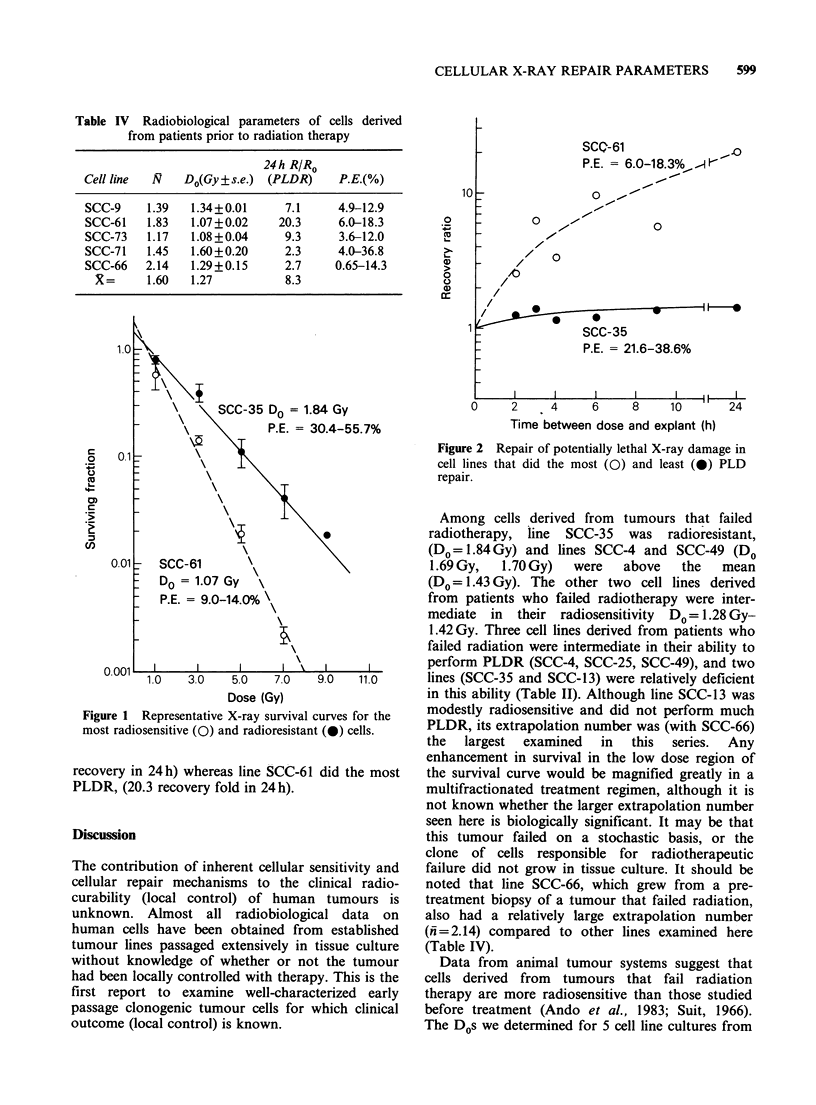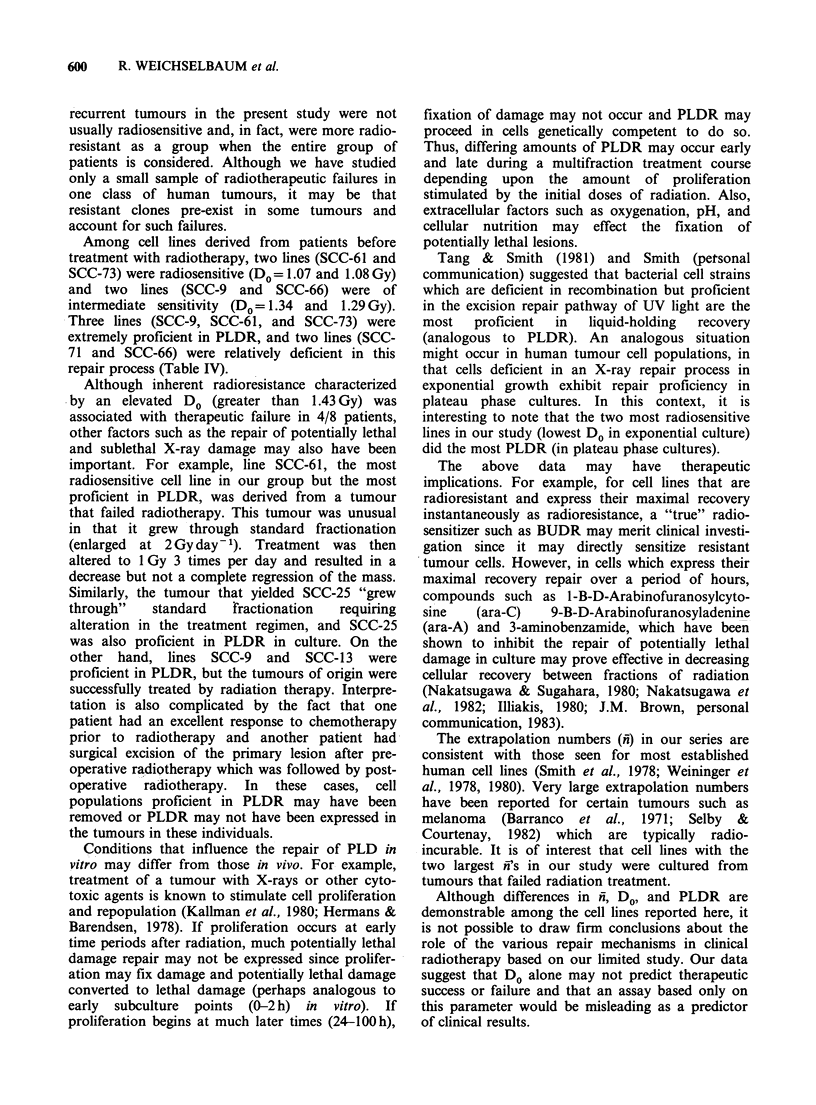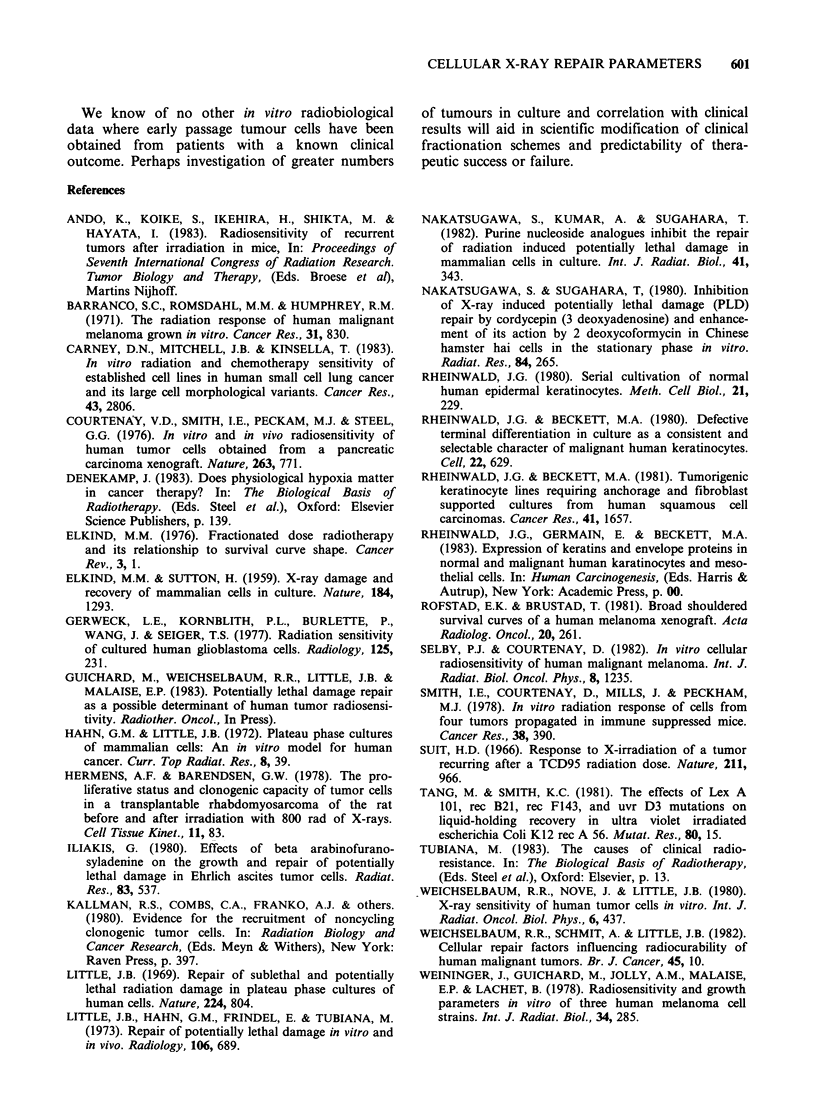# Cellular X-ray repair parameters of early passage squamous cell carcinoma lines derived from patients with known responses to radiotherapy.

**DOI:** 10.1038/bjc.1984.94

**Published:** 1984-05

**Authors:** R. Weichselbaum, W. Dahlberg, J. B. Little, T. J. Ervin, D. Miller, S. Hellman, J. G. Rheinwald

## Abstract

We have investigated X-ray survival parameters and repair of potentially lethal damage ( PLDR ) in ten early passage squamous cell carcinoma cell lines derived from patients who were biopsied before initiation of radiotherapy or after radiation therapy failure. Radiosensitivity (D0) ranged from 1.07 to 1.93 (Gy), extrapolation numbers (-n) from 1.17 to 2.14 and PLD recovery at 24 h from 1.4 to 20.3. Despite significant differences in these parameters amongst the cell lines, a firm correlation between radiocurability and any individual radiobiological parameter could not be established. Our data suggest that the mechanisms associated with radioresistance are complex and that any single radiobiological parameter may not predict clinical success or failure.


*Br. J. Cancer* (1984), **49**, 595–601

### Cellular X-ray repair parameters of early passage squamous cell carcinoma lines derived from patients with known responses to radiotherapy

R. Weichselbaum[1], W. Dahlberg[1], J.B. Little[1], T.J. Ervin[2], D. Miller[2], S. Hellman[3]*& J.G. Rheinwald[4]

[1]*Laboratory of Radiobiology, Department of Cancer Biology, Harvard School of Public Health and Joint Center for Radiation Therapy, Department of Radiation Therapy,* [2]*Divisions of Medicine and Surgery, Dana-Farber Cancer Institute, and Harvard Medical School, Boston, MA,* [3]*Joint Center for Radiation Therapy, Department of Radiation Therapy,* [4]*Division of Cell Growth and Regulation, Dana-Farber Cancer Institute, Department of Physiology and Biophysics, Harvard Medical School, Boston, MA, U.S.A.*

**Summary** We have investigated X-ray survival parameters and repair of potentially lethal damage (PLDR) in ten early passage squamous cell carcinoma cell lines derived from patients who were biopsied before initiation of radiotherapy or after radiation therapy failure.

Radiosensitivity ($D_0$) ranged from 1.07 to 1.93 (Gy), extrapolation numbers ($\bar{n}$) from 1.17 to 2.14 and PLD recovery at 24 h from 1.4 to 20.3. Despite significant differences in these parameters amongst the cell lines, a firm correlation between radiocurability and any individual radiobiological parameter could not be established. Our data suggest that the mechanisms associated with radioresistance are complex and that any single radiobiological parameter may not predict clinical success or failure.

Ionizing radiation has become an integral part of human cancer therapy, although the biological explanation for therapeutic success or failure remains elusive. Radiotherapy delivered in multiple small doses (1.5–3.0 Gy day$^{-1}$) has been found to have a higher therapeutic ratio than radiation delivered as a large single dose (Tubiana, 1983). Attempts to explain the advantage of fractionation as well as the cause of failure of radiation treatment in certain clinical circumstances have invoked a variety of mechanisms. These include redistribution of cells within the cell cycle following radiation, the presence of hypoxic tumour cells, and the reoxygenation of these cells. While investigation of the latter two factors has yielded interesting information in animal tumour systems, cell hypoxia as the major determinant of radiation failure in human cancer has not been established (Denekamp, 1983).

Another area of interest in radiobiology is the study of the intrinsic X-ray sensitivity or resistance of tumour cells and the repair of sublethal and potentially lethal X-ray damage. It has been demonstrated that when a single dose of X-rays is divided into two fractions separated by an interval of several hours, an enhancement in survival occurs. This split dose recovery phenomenon has been interpreted as reflecting the repair of sublethal radiation damage induced by the first dose in cells that survive this dose (Elkind, 1959). The magnitude of this effect can be expressed by the extrapolation number ($\bar{n}$) which is the back extrapolation of the slope to the ordinate (the shoulder of the survival curve); the shoulder is thought to represent the ability of cells to accumulate sublethal X-ray injury (Elkind, 1976).

When monolayer cultures of mammalian cells are maintained under conditions of constant medium renewal without subculture, they enter a crowded, density-inhibited state of growth in which the fraction of dividing cells is reduced and a large population of nonproliferating cells accumulates (Little, 1969). This is an experimental condition which may resemble the physiological state of tumour cell populations *in vivo* since these may contain a large population of nondividing but potentially clonogenic cells. When such plateau phase cultures are treated with X-rays or chemical agents and subculture of the cells is delayed, an enhancement in survival occurs. This phenomenon has been referred to as recovery from potentially lethal X-ray damage, and may be analogous to liquid-holding recovery in bacteria and yeast (Little, 1969; Hahn & Little, 1972). PLDR has been described in experimental solid and ascites tumours as well as in established human tumour cell lines

*Present address: Memorial Sloan-Kettering Cancer Center, New York, U.S.A.

Correspondence: R.W. Weichselbaum

Received 11 November 1983; accepted 2 February 1984.

© The Macmillan Press Ltd., 1984

(Little *et al.*, 1973; Weichselbaum *et al.*, 1982; Guichard *et al.*, in press).

Experimental evidence indicates that many established human tumour cell lines in culture are not intrinsically more sensitive or resistant to the lethal effects of X-rays than are cells obtained from normal tissues (Weichselbaum *et al.*, 1980; Smith *et al.*, 1978; Weininger *et al.*, 1978). Exceptions have been reported, however; Weichselbaum *et al.* (1982) described an inherently radioresistant melanoma line and Gerweck *et al.* (1977) and Nilsson *et al.* (1980) reported several radioresistant glioblastoma lines. Unusual repair parameters have been reported in some human tumor cell lines as well. For example, Barranco *et al.* (1971) reported melanoma lines with large shoulders ($\bar{n}$), and Carney *et al.* (1983) investigated two large cell lung carcinoma lines with relatively large extrapolation numbers, although much smaller than those reported by Barranco *et al.* (1971). for their melanoma lines. Selby & Courtenay (1982) reported a large shoulder for two human melanoma xenografts grown in agar diffusion chambers. Courtenay *et al.* (1976) found a xenografted human pancreatic carcinoma proficient in the repair of potentially lethal damage. Weichselbaum *et al.* (1982) studied two human melanoma cell lines and one osteosarcoma line which were especially proficient in the repair of potentially lethal X-ray damage. Both groups suggested that one factor in the failure of X-rays to sterilize a malignant tumour could be the ability of noncycling cells to recover from potentially lethal damage. Rofstad & Brustad (1981) reported a human melanoma line proficient in both sublethal and potentially lethal damage repair. It should be noted, however, that all of these studies were carried out on cell lines passaged many times *in vitro*, and for none was the clinical outcome of the patient from whom the line was derived known.

In order to determine the possible contribution of cellular radiosensitivity, sublethal and potentially lethal damage repair in head and neck cancer therapy, we studied 10 early passage tumour cell populations derived from patients with head and neck squamous cell carcinoma. Five biopsies were obtained from patients before the institution of therapy and 5 from patients who suffered radiation failures. Our study is unique in that cell populations from each tumour were serially cultivated under identical conditions and were studied between 10 and 15 passages after initial explant, and correlation with clinical (radiocurability) results was possible. We determined X-ray survival parameters including $\bar{n}$ and $D_0$ as well as repair of potentially lethal X-ray damage for these tumours in culture.

### Materials and methods

### *Isolation of tumour cells*

Methods of establishment and characterization of squamous cell carcinoma lines have been published (Rheinwald & Beckett, 1980, 1981) and are briefly summarized here. Biopsies of squamous cell carcinoma were obtained from patients seen in the multidisciplinary head and neck tumour clinic at the Dana-Farber Cancer Institute (DFCI) and the Joint Center for Radiation Therapy (JCRT). Culture conditions and procedures were similar to those for preparing keratinocyte cultures from normal skin, including co-culture with a 3T3 fibroblast feeder layer (Rheinwald, 1980). Biopsies were placed immediately into culture medium within 2 h of removal. Samples were rinsed with serum-free medium containing penicillin or streptomycin and cut into pieces 3 mm in diameter. A portion was sectioned and stained with Haematoxylin and Eosin in order to confirm that the biopsies contained squamous cell carcinoma. The remaining fragments were minced with scissors into pieces <1 mm in diameter and were distributed to culture dishes and held to the surface with a small plasma clot. One day after plating, mitomycin C-treated, Swiss mouse embryonic fibroblast 3T3 cells were added as a feeder layer.

Growth medium consisted of Dulbecco's modified Eagle's medium, 20% foetal calf serum, and $0.4\,\mu g\,ml^{-1}$ hydrocortisone. Primary cultures were subcultured after 1–2 weeks, at which time individual explant colonies had attained a diameter of 0.5 cm–1.0 cm, and before neighboring colonies had merged to make a confluent monolayer. Tumour cell populations were disaggregated by a 15–30 min incubation with 0.05% Trypsin plus 0.02% EDTA at 37° and were serially passaged at 7–10 day intervals by subculturing preconfluent cultures that had been initiated from 1–$3\times10^4$ cells/60 ml dish together with 3T3 feeder cells. Each passage was equivalent to about 7–10 cell generations. As reported previously (Rheinwald & Beckett, 1981), the tumour lines retained unique aneuploid karyotypes and distinctive morphological characteristics indefinitely from the first passage, suggesting that the lines represent the major stem cell population of their respective tumours. Tumours from the oral cavity grew with a higher frequency of success than from other head and neck sites (Rheinwald *et al.*, 1983).

### *Radiation experiments*

X-ray survival curves were determined as follows. Cells at the 8th to 10th passages were maintained in medium without 3T3 cells at 37°C in a humidified atmosphere of 5% $CO_2$ in air. Cells were trypsinized with 0.05% trypsin from stock cultures and between 500 and 40,000 cells were plated in 10 cm diameter dishes and allowed to enter exponential growth. Radiation was carried out 18 h later with a GE Maximar X-ray generator at 220 Kvp and 15 MA yielding a dose rate of 0.8 Gy $min^{-1}$. Immediately after radiation the cultures were returned to the incubator. After 18–24 days, the cells were fixed and stained with Crystal violet. Only colonies of $\geqslant 50$ cells were scored as survivors. All data points are the results of 2–4 experiments. Radiation survival curve parameters measured are the $D_0$, which is the inverse of the slope of the radiation survival curve, and the extrapolation number ($\bar{n}$) which is the back extrapolation of the slope to the ordinate. These parameters were determined by a least squares regression analysis of all data points.

PLDR studies were performed as follows. Cells were initially seeded into 60 mm plastic petri dishes and grown to confluency. Culture medium was renewed daily for 3 days and experiments performed on the fourth. Cells were irradiated at room temperature and afterwards were returned to the incubator. Single dishes were removed and cells subcultured and seeded at low density (10,000–80,000 cells) at regular intervals thereafter.

Seven Gy was used to study PLD recovery. Initial (0 h subculture) surviving fractions were similar in 8 of the 10 cell lines. SCC-61 and SCC-73 were exceptions and showed lower initial surviving fractions than the other cell lines studied. The enhancement in survival, as measured by the factor of increased colony-forming ability resulting from delay in subculture after irradiation, is interpreted as being due to the repair of potentially lethal damage. PLDR is expressed in terms of enhancement in surviving fraction as a function of time interval between radiation and subculture after a single dose of radiation and is expressed as a recovery ratio ($R/R_0$) by dividing the 24 h surviving fraction (R) by the 0 h surviving fraction ($R_0$). Although growth of some of the cell lines was greatly enhanced by the use of feeder layer support, survival curve parameters were independent of the presence of a feeder layer. Feeder layers used in radiation experiments were reproductively inactivated with 100 Gy from a 2Ci Cobalt-60 source.

### Results

Table I shows a summary of clinical stage, site, and response to radiotherapy in patients who suffered local radiation failures. All patients had completed a course of radiation therapy undertaken with

**Table I** Cell lines derived from tumours that failed fractionated radiotherapy

| *Line* | *Stage* | *Site* | *Response to radiotherapy* | *Comments* |
|---|---|---|---|---|
| SCC-4 | $T_3N_0$ | floor of mouth | little response persistent tumour | in-field persistence |
| SCC-25 | $T_2N_1$ | oral tongue | grew through radiotherapy treatment aborted at 3.4 Gy | unusually aggressive tumour patient treated with 2.0 Gy twice per day no effect on rapidly increasing tumour size |
| SCC-35 | $T_4N_0$ | pyriform sinus | complete response to RT | in-field recurrence 2 years later |
| SCC-13 | $T_2N_0$ | skin of face | complete response to RT | recurrence 5 months later |
| SCC-49 | $T_2N_0$ | tonsil | partial response | in-field recurrence 14 months later |

curative intent. Portal films or charts were reviewed when available to certify that tumours were in-field failures and not marginal recurrences, the result of technical errors or excessively protracted fractionation. Table II shows $\bar{n}$, $D_0$, plating efficiency and 24 h recovery ratio (PLDR) in tumour cells derived from patients who suffered local radiation failures. Twenty-four hour recovery ratio ($R/R_0$) represents the amount of PLDR performed by each cell line $D_0$'s (radiosensitivity) ranged from 1.20–1.84 Gy (mean 1.58 Gy). Extrapolation numbers ($\bar{n}$) ranged from 1.49–2.11 (mean 1.66). PLD recovery ratio ranged from 1.4–6.2.

**Table II** Radiobiological parameters of cells from patients who failed radiation therapy

| *Cell line* | $\bar{N}$ | $D_0(Gy \pm s.e.)$ | *24 h R/R$_0$ (PLDR)* | *P.E.(%)* |
|---|---|---|---|---|
| SCC-4 | 1.49 | 1.69±0.15 | 4.4 | 8.5–15.2 |
| SCC-25 | 1.53 | 1.42±0.01 | 6.2 | 7.2–17.8 |
| SCC-35 | 1.63 | 1.84±0.19 | •1.4 | 21.6–55.7 |
| SCC-13 | 2.11 | 1.28±0.07 | 2.2 | 13.8–19.1 |
| SCC-49 | 1.55 | 1.70±0.12 | 4.9 | 11.7–17.2 |
| $\bar{X}$= | 1.66 | 1.58 | 3.8 | |

Table III shows a summary of clinical stage, site and response to radiotherapy as well as local control (radiocurability) in patients who had a biopsy prior to radiotherapy delivered with curative intent. One patient had chemotherapy and one patient had surgery prior to the initiation of radiation. Local control results were assessed in the multidisciplinary JCRT-DFCI head and neck clinic and recurrent tumours were proven by biopsy. One patient died of a myocardial infarction; he showed no histological evidence of tumour at autopsy.

Table IV shows a summary of $\bar{n}$, $D_0$ (radiosensitivity) plating efficiencies, and 24 h recovery ratio (PLDR) in cells derived from tumours in which biopsy was obtained before initiation of treatment. $D_0$s ranged from 1.07–1.60 Gy with a mean of 1.27 Gy and $n$ ranged from 1.17–2.14 with a mean of 1.60. The 24 h PLD recovery ratio ranged from 2.7–20.3 with a mean of 8.1. The mean $D_0$ for all 10 cell lines was 1.43 Gy and the mean $n$ was 1.63.

Figure 1 shows representative X-ray survival curves for the most radiosensitive and radioresistant cells in our study. SCC-35 was the most resistant ($D_0$=1.84 Gy) whereas SCC-61 was the most radiosensitive ($D_0$=1.07 Gy). Figure 2 shows the repair of potentially lethal X-ray damage in cell lines that did the most and least PLD repair in our study. Line SCC-35 did the least PLDR, (1.4 fold

**Table III** Cell lines derived from tumours before initiation of radiotherapy

| *Line* | *Stage* | *Site* | *Response to radiotherapy* | *Comments* |
|---|---|---|---|---|
| SCC-9 | $T_2N_1$ | oral tongue | 4.5 Gy pre-op<br>2.0 Gy post-op<br>complete response prior to surgery | no tumour in surgical specimen<br>died of distant disease<br>local control<br>no tumour at autopsy |
| SCC-61 | $T_{(4x)}N_{(2Bx)}$ | oral tongue | partial response with almost complete regression after change in fractionation | unusually aggressive tumour enlarged on standard fractionation treatment. Changed to 1 Gy 3 times a day |
| SCC-73 | $T_4N_0$ | retromolar trigone | 6.84 Gy post-op | good partial response to chemotherapy prior to RT local and distant control<br>died result of myocardial infarction<br>no tumour at autopsy |
| SCC-71 | $T_4N_1$ | soft pallate | some response but persistent disease | persistent disease |
| SCC-66 | $T_{4x}N_0$ | floor of mouth | partial response to radiation therapy, but never disease free | persistent disease |

**Table IV** Radiobiological parameters of cells derived from patients prior to radiation therapy

| *Cell line* | $\bar{N}$ | $D_0(Gy \pm s.e.)$ | *24 h* $R/R_0$ *(PLDR)* | *P.E.(%)* |
|---|---|---|---|---|
| SCC-9 | 1.39 | 1.34±0.01 | 7.1 | 4.9–12.9 |
| SCC-61 | 1.83 | 1.07±0.02 | 20.3 | 6.0–18.3 |
| SCC-73 | 1.17 | 1.08±0.04 | 9.3 | 3.6–12.0 |
| SCC-71 | 1.45 | 1.60±0.20 | 2.3 | 4.0–36.8 |
| SCC-66 | 2.14 | 1.29±0.15 | 2.7 | 0.65–14.3 |
| $\bar{X}$= | 1.60 | 1.27 | 8.3 | |

**Figure 1** Representative X-ray survival curves for the most radiosensitive (○) and radioresistant (●) cells.

recovery in 24 h) whereas line SCC-61 did the most PLDR, (20.3 recovery fold in 24 h).

### Discussion

The contribution of inherent cellular sensitivity and cellular repair mechanisms to the clinical radiocurability (local control) of human tumours is unknown. Almost all radiobiological data on human cells have been obtained from established tumour lines passaged extensively in tissue culture without knowledge of whether or not the tumour had been locally controlled with therapy. This is the first report to examine well-characterized early passage clonogenic tumour cells for which clinical outcome (local control) is known.

**Figure 2** Repair of potentially lethal X-ray damage in cell lines that did the most (○) and least (●) PLD repair.

Among cells derived from tumours that failed radiotherapy, line SCC-35 was radioresistant, ($D_0$=1.84 Gy) and lines SCC-4 and SCC-49 ($D_0$ 1.69 Gy, 1.70 Gy) were above the mean ($D_0$=1.43 Gy). The other two cell lines derived from patients who failed radiotherapy were intermediate in their radiosensitivity $D_0$=1.28 Gy–1.42 Gy. Three cell lines derived from patients who failed radiation were intermediate in their ability to perform PLDR (SCC-4, SCC-25, SCC-49), and two lines (SCC-35 and SCC-13) were relatively deficient in this ability (Table II). Although line SCC-13 was modestly radiosensitive and did not perform much PLDR, its extrapolation number was (with SCC-66) the largest examined in this series. Any enhancement in survival in the low dose region of the survival curve would be magnified greatly in a multifractionated treatment regimen, although it is not known whether the larger extrapolation number seen here is biologically significant. It may be that this tumour failed on a stochastic basis, or the clone of cells responsible for radiotherapeutic failure did not grow in tissue culture. It should be noted that line SCC-66, which grew from a pretreatment biopsy of a tumour that failed radiation, also had a relatively large extrapolation number ($\bar{n}$=2.14) compared to other lines examined here (Table IV).

Data from animal tumour systems suggest that cells derived from tumours that fail radiation therapy are more radiosensitive than those studied before treatment (Ando *et al.*, 1983; Suit, 1966). The $D_0$s we determined for 5 cell line cultures from recurrent tumours in the present study were not usually radiosensitive and, in fact, were more radioresistant as a group when the entire group of patients is considered. Although we have studied only a small sample of radiotherapeutic failures in one class of human tumours, it may be that resistant clones pre-exist in some tumours and account for such failures.

Among cell lines derived from patients before treatment with radiotherapy, two lines (SCC-61 and SCC-73) were radiosensitive ($D_0 = 1.07$ and 1.08 Gy) and two lines (SCC-9 and SCC-66) were of intermediate sensitivity ($D_0 = 1.34$ and 1.29 Gy). Three lines (SCC-9, SCC-61, and SCC-73) were extremely proficient in PLDR, and two lines (SCC-71 and SCC-66) were relatively deficient in this repair process (Table IV).

Although inherent radioresistance characterized by an elevated $D_0$ (greater than 1.43 Gy) was associated with therapeutic failure in 4/8 patients, other factors such as the repair of potentially lethal and sublethal X-ray damage may also have been important. For example, line SCC-61, the most radiosensitive cell line in our group but the most proficient in PLDR, was derived from a tumour that failed radiotherapy. This tumour was unusual in that it grew through standard fractionation (enlarged at 2 Gy day$^{-1}$). Treatment was then altered to 1 Gy 3 times per day and resulted in a decrease but not a complete regression of the mass. Similarly, the tumour that yielded SCC-25 "grew through" standard fractionation requiring alteration in the treatment regimen, and SCC-25 was also proficient in PLDR in culture. On the other hand, lines SCC-9 and SCC-13 were proficient in PLDR, but the tumours of origin were successfully treated by radiation therapy. Interpretation is also complicated by the fact that one patient had an excellent response to chemotherapy prior to radiotherapy and another patient had surgical excision of the primary lesion after preoperative radiotherapy which was followed by postoperative radiotherapy. In these cases, cell populations proficient in PLDR may have been removed or PLDR may not have been expressed in the tumours in these individuals.

Conditions that influence the repair of PLD *in vitro* may differ from those *in vivo*. For example, treatment of a tumour with X-rays or other cytotoxic agents is known to stimulate cell proliferation and repopulation (Kallman *et al.*, 1980; Hermans & Barendsen, 1978). If proliferation occurs at early time periods after radiation, much potentially lethal damage repair may not be expressed since proliferation may fix damage and potentially lethal damage converted to lethal damage (perhaps analogous to early subculture points (0–2 h) *in vitro*). If proliferation begins at much later times (24–100 h), fixation of damage may not occur and PLDR may proceed in cells genetically competent to do so. Thus, differing amounts of PLDR may occur early and late during a multifraction treatment course depending upon the amount of proliferation stimulated by the initial doses of radiation. Also, extracellular factors such as oxygenation, pH, and cellular nutrition may effect the fixation of potentially lethal lesions.

Tang & Smith (1981) and Smith (personal communication) suggested that bacterial cell strains which are deficient in recombination but proficient in the excision repair pathway of UV light are the most proficient in liquid-holding recovery (analogous to PLDR). An analogous situation might occur in human tumour cell populations, in that cells deficient in an X-ray repair process in exponential growth exhibit repair proficiency in plateau phase cultures. In this context, it is interesting to note that the two most radiosensitive lines in our study (lowest $D_0$ in exponential culture) did the most PLDR (in plateau phase cultures).

The above data may have therapeutic implications. For example, for cell lines that are radioresistant and express their maximal recovery instantaneously as radioresistance, a "true" radiosensitizer such as BUDR may merit clinical investigation since it may directly sensitize resistant tumour cells. However, in cells which express their maximal recovery repair over a period of hours, compounds such as 1-B-D-Arabinofuranosylcytosine (ara-C) 9-B-D-Arabinofuranosyladenine (ara-A) and 3-aminobenzamide, which have been shown to inhibit the repair of potentially lethal damage in culture may prove effective in decreasing cellular recovery between fractions of radiation (Nakatsugawa & Sugahara, 1980; Nakatsugawa *et al.*, 1982; Illiakis, 1980; J.M. Brown, personal communication, 1983).

The extrapolation numbers ($\bar{n}$) in our series are consistent with those seen for most established human cell lines (Smith *et al.*, 1978; Weininger *et al.*, 1978, 1980). Very large extrapolation numbers have been reported for certain tumours such as melanoma (Barranco *et al.*, 1971; Selby & Courtenay, 1982) which are typically radioincurable. It is of interest that cell lines with the two largest $\bar{n}$'s in our study were cultured from tumours that failed radiation treatment.

Although differences in $\bar{n}$, $D_0$, and PLDR are demonstrable among the cell lines reported here, it is not possible to draw firm conclusions about the role of the various repair mechanisms in clinical radiotherapy based on our limited study. Our data suggest that $D_0$ alone may not predict therapeutic success or failure and that an assay based only on this parameter would be misleading as a predictor of clinical results.

We know of no other *in vitro* radiobiological data where early passage tumour cells have been obtained from patients with a known clinical outcome. Perhaps investigation of greater numbers of tumours in culture and correlation with clinical results will aid in scientific modification of clinical fractionation schemes and predictability of therapeutic success or failure.

**References**

ANDO, K., KOIKE, S., IKEHIRA, H., SHIKTA, M. & HAYATA, I. (1983). Radiosensitivity of recurrent tumors after irradiation in mice, In: *Proceedings of Seventh International Congress of Radiation Research. Tumor Biology and Therapy,* (Eds. Broese *et al*), Martins Nijhoff.

BARRANCO, S.C., ROMSDAHL, M.M. & HUMPHREY, R.M. (1971). The radiation response of human malignant melanoma grown *in vitro*. *Cancer Res.*, **31,** 830.

CARNEY, D.N., MITCHELL, J.B. & KINSELLA, T. (1983). *In vitro* radiation and chemotherapy sensitivity of established cell lines in human small cell lung cancer and its large cell morphological variants. *Cancer Res.*, **43,** 2806.

COURTENAY, V.D., SMITH, I.E., PECKAM, M.J. & STEEL, G.G. (1976). *In vitro* and *in vivo* radiosensitivity of human tumor cells obtained from a pancreatic carcinoma xenograft. *Nature,* **263,** 771.

DENEKAMP, J. (1983). Does physiological hypoxia matter in cancer therapy? In: *The Biological Basis of Radiotherapy*. (Eds. Steel *et al.*), Oxford: Elsevier Science Publishers, p. 139.

ELKIND, M.M. (1976). Fractionated dose radiotherapy and its relationship to survival curve shape. *Cancer Rev.*, **3,** 1.

ELKIND, M.M. & SUTTON, H. (1959). X-ray damage and recovery of mammalian cells in culture. *Nature,* **184,** 1293.

GERWECK, L.E., KORNBLITH, P.L., BURLETTE, P., WANG, J. & SEIGER, T.S. (1977). Radiation sensitivity of cultured human glioblastoma cells. *Radiology,* **125,** 231.

GUICHARD, M., WEICHSELBAUM, R.R., LITTLE, J.B. & MALAISE, E.P. (1983). Potentially lethal damage repair as a possible determinant of human tumor radiosensitivity. *Radiother. Oncol.*, In Press).

HAHN, G.M. & LITTLE, J.B. (1972). Plateau phase cultures of mammalian cells: An *in vitro* model for human cancer. *Curr. Top Radiat. Res.*, **8,** 39.

HERMENS, A.F. & BARENDSEN, G.W. (1978). The proliferative status and clonogenic capacity of tumor cells in a transplantable rhabdomyosarcoma of the rat before and after irradiation with 800 rad of X-rays. *Cell Tissue Kinet.*, **11,** 83.

ILIAKIS, G. (1980). Effects of beta arabinofuranosyladenine on the growth and repair of potentially lethal damage in Ehrlich ascites tumor cells. *Radiat. Res.*, **83,** 537.

KALLMAN, R.S., COMBS, C.A., FRANKO, A.J. & others. (1980). Evidence for the recruitment of noncycling clonogenic tumor cells. In: *Radiation Biology and Cancer Research,* (Eds. Meyn & Withers), New York: Raven Press, p. 397.

LITTLE, J.B. (1969). Repair of sublethal and potentially lethal radiation damage in plateau phase cultures of human cells. *Nature,* **224,** 804.

LITTLE, J.B., HAHN, G.M., FRINDEL, E. & TUBIANA, M. (1973). Repair of potentially lethal damage *in vitro* and *in vivo*. *Radiology,* **106,** 689.

NAKATSUGAWA, S., KUMAR, A. & SUGAHARA, T. (1982). Purine nucleoside analogues inhibit the repair of radiation induced potentially lethal damage in mammalian cells in culture. *Int. J. Radiat. Biol.*, **41,** 343.

NAKATSUGAWA, S. & SUGAHARA, T, (1980). Inhibition of X-ray induced potentially lethal damage (PLD) repair by cordycepin (3 deoxyadenosine) and enhancement of its action by 2 deoxycoformycin in Chinese hamster hai cells in the stationary phase *in vitro*. *Radiat. Res.*, **84,** 265.

RHEINWALD, J.G. (1980). Serial cultivation of normal human epidermal keratinocytes. *Meth. Cell Biol.*, **21,** 229.

RHEINWALD, J.G. & BECKETT, M.A. (1980). Defective terminal differentiation in culture as a consistent and selectable character of malignant human keratinocytes. *Cell,* **22,** 629.

RHEINWALD, J.G. & BECKETT, M.A. (1981). Tumorigenic keratinocyte lines requiring anchorage and fibroblast supported cultures from human squamous cell carcinomas. *Cancer Res.*, **41,** 1657.

RHEINWALD, J.G., GERMAIN, E. & BECKETT, M.A. (1983). Expression of keratins and envelope proteins in normal and malignant human karatinocytes and mesothelial cells. In: *Human Carcinogenesis,* (Eds. Harris & Autrup), New York: Academic Press, p. **00.**

ROFSTAD, E.K. & BRUSTAD, T. (1981). Broad shouldered survival curves of a human melanoma xenograft. *Acta Radiolog. Oncol.*, **20,** 261.

SELBY, P.J. & COURTENAY, D. (1982). *In vitro* cellular radiosensitivity of human malignant melanoma. *Int. J. Radiat. Biol. Oncol. Phys.*, **8,** 1235.

SMITH, I.E., COURTENAY, D., MILLS, J. & PECKHAM, M.J. (1978). *In vitro* radiation response of cells from four tumors propagated in immune suppressed mice. *Cancer Res.*, **38,** 390.

SUIT, H.D. (1966). Response to X-irradiation of a tumor recurring after a TCD95 radiation dose. *Nature,* **211,** 966.

TANG, M. & SMITH, K.C. (1981). The effects of Lex A 101, rec B21, rec F143, and uvr D3 mutations on liquid-holding recovery in ultra violet irradiated escherichia Coli K12 rec A 56. *Mutat. Res.*, **80,** 15.

TUBIANA, M. (1983). The causes of clinical radioresistance. In: *The Biological Basis of Radiotherapy,* (Eds. Steel *et al.*), Oxford: Elsevier, p. 13.

WEICHSELBAUM, R.R., NOVE, J. & LITTLE, J.B. (1980). X-ray sensitivity of human tumor cells *in vitro*. *Int. J. Radiat. Oncol. Biol. Phys.*, **6,** 437.

WEICHSELBAUM, R.R., SCHMIT, A. & LITTLE, J.B. (1982). Cellular repair factors influencing radiocurability of human malignant tumors. *Br. J. Cancer,* **45,** 10.

WEININGER, J., GUICHARD, M., JOLLY, A.M., MALAISE, E.P. & LACHET, B. (1978). Radiosensitivity and growth parameters *in vitro* of three human melanoma cell strains. *Int. J. Radiat. Biol.*, **34,** 285.